# Polymorphisms in the *ASAP1* and *SP110* Genes and Its Association with the Susceptibility to Pulmonary Tuberculosis in a Mongolian Population

**DOI:** 10.1155/2022/2713869

**Published:** 2022-09-20

**Authors:** Xiaogang Cui, Tianqi Yuan, Pengyuan Ning, Jie Han, Yue Liu, Jiao Feng, Fan Lian, Mingyuan Hao, Li Dong, Jinqi Hao, Li Xing, Changxin Wu

**Affiliations:** ^1^Key Lab of Medical Molecular Cell Biology of Shanxi Province, Taiyuan 030006, China; ^2^Key Laboratory of Chemical Biology and Molecular Engineering of Ministry of Education; Shanxi Provincial Key Laboratory for Major Infectious Disease Response, Institutes of Biomedical Sciences, Shanxi University, Taiyuan 030006, China; ^3^The Second People's Hospital in Hulunbuir, Zaerdong 162650, China; ^4^Baotou Medical College, Inner Mongolia University of Science and Technology, No. 31, Jianshe Road, Donghe District, Baotou 014060, China

## Abstract

Tuberculosis (TB) remains one of the deadliest infectious diseases in the world. Previous genome-wide association studies suggested that single-nucleotide polymorphisms (SNPs) in some genes could indicate the susceptibility to TB in some populations. Herein, we studied the association of SNPs in the immunity-related genes, i.e., *ASAP1* and *SP110* genes with the susceptibility to TB in a Mongolian population in China. A case–control study was performed with 197 TB patients and 217 healthy controls. Six SNPs in *ASAP1* and six SNPs in *SP110* were selected for genotyping test by second-generation sequencing technique. A SNP in *SP110* gene (rs722555) was identified to be associated with susceptibility to TB in the Mongolian population (*p* < 0.05). The T allele of rs722555 in *SP110* gene was associated with a 36% increase of risk at TB (OR 1.36, 95% CI 1.03–1.81), and the CT+TT genotype of rs722555 was associated with a 74% increase of risk at TB (OR 1.74, 95% CI 1.16–2.60) in the dominant genetic model. None of SNPs in *ASAP1* gene tested in this study were significantly associated with TB susceptibility, while some individuals with SNPs (rs10956514, rs4733781, rs2033059, rs12680942, rs1017281, rs1469288, and rs17285138) in the *ASAP1* gene tended to have a reduced risk at TB. In conclusion, this study suggested that the rs722555 SNP in *SP110* gene might be a risk factor for TB in a Mongolian population.

## 1. Introduction

Tuberculosis (TB), caused by *Mycobacterium tuberculosis* bacillus (Mtb), is one of the oldest infectious diseases. According to the newest data from World Health Organization (WHO), around 9.9 million (range: 9–11 million) people became newly sick with TB in 2021, 86% of which (about 7–8 million) resided in 30 high-burden countries [1]. However, only 4–6 million were officially diagnosed. Between 1 and 3 million people are estimated to die from TB each year [1]. Previous epidemiological studies have reported that almost 25% of the population are latently infected with Mtb, but only 5% of these individuals might develop into the active disease during their lifetime [2], indicating that TB is a multifactorial disease and its development is affected by many factors [3, 4].

Genetic factors, in addition to malnutrition, human immunodeficiency, virus infection, and environmental factors, have been documented to influence the risk of TB [3, 4]. For genetic factors, a large number of studies have investigated the association between genetic polymorphisms and the risk of TB, in which the genes encoding Arf-GAP with SH3 domain, ankyrin repeat, and PH domain 1 (*ASAP1*) and Speckled 110 (*SP110*) are the most striking [3–14]. *ASAP1* is also known as *AMAP1* or *DDEF1*. It encodes *ASAP1*, a member of ADP-ribosylation factor GTPase-activating proteins (Arf-GAPs), which is a multifunctional scaffold protein [9, 15]. The expression of *ASAP1* regulates the cell motility and invasion and also affects the progression and metastasis of tumor cells including ovary cancer [16], prostate cancer [17], and breast cancer [18, 19]. To screen the genes that exert the largest impact on susceptibility to a multifactorial disease at a population level [20], the genome-wide association studies (GWAS) [21] have identified *ASAP1* as a novel gene associated with the susceptibility to TB. Two SNPs (rs4733781 and rs10956514) in the *ASAP1* gene are significantly associated with susceptibility to TB in a Russian population [5]. Moreover, *ASAP1* expression is markedly decreased in Mtb-infected dendritic cells, which may result in impaired dendritic cell migration and indicate a potential mechanism of *ASAP1* polymorphisms to predispose individuals to TB.

*SP110* gene could also affect the susceptibility to TB. *SP110* is the human homolog of the intracellular pathogen resistance-1 (*Ipr1*) gene in mice. *Ipr1* is located on chromosome 1 at the supersusceptibility to tuberculosis 1 (*sst1*) locus and associated with resistance to pulmonary TB in a murine model [7]. *SP110-*encoded protein is a component of nuclear bodies [4]. This protein can mediate interactions between hosts and pathogens by participating in the activation of the response to intracellular pathogens in macrophages at the transcriptional level. In 2006, Tosh et al. first reported that *SP110* was related to TB in a West African population using a family-based experimental design. Subsequently, more studies with various study designs were conducted and demonstrated the associations of polymorphisms in *SP110* with LTBI susceptibility [4, 5, 7, 8, 10–13].

To understand if the population diversity and genetic heredity could influence the association of SNPs in *ASAP1* and *SP110* with the susceptibility of TB, we selected a set of SNPs and focused on genetic polymorphisms relating to pulmonary TB in a minority Mongolian population in China. The clinical relevance of SNPs in these two genes to the development of pulmonary TB was explored.

## 2. Materials and Methods

### 2.1. Study Population and Sample Collection

A total of 414 participants were involved in this study, consisting of 197 active TB patients as the cases and 217 healthy volunteers as the controls. The cases were diagnosed with TB for the first time and recruited from the Second People's Hospital of Hulunbuir city, Heilongjiang province, China, where the Mongolian people lived for several generations. Pulmonary tuberculosis (PTB) patients were diagnosed based on the clinical information including positive Mtb sputum culture, sputum smear analysis with acid-fast bacillus microscopy, clinical symptoms, and X-ray or CT scanning and histological pathology. The volunteers were healthy blood donors who had negative results for the TB-interferon gamma release assay (TB-IGRA), no history of TB infection, and were normal in physical examinations. In this study, all individuals were from the minority Mongolian population. The demographic and primary clinical data were obtained by interviewing the participants and/or retrieved from their hospital medical records with permission.

This research involving human subjects complied with all relevant national regulations and institutional policies and was in accordance with the tenets of the Helsinki Declaration (revised in 2013). This study was approved by the Research Ethics Committee of Baotou Medical College, Inner Mongolia University of Science and Technology (no. 2018002).

### 2.2. Selection of SNPs and Genotyping

Genetic variation data for candidate SNPs in *ASAP1* and *SP110* were obtained via a thorough scan of the dbSNP database (https://www.ncbi.nlm.nih.gov/snp/). The SNPs within potentially functional regions (i.e., exon, promoter, or untranslated regions) were selected. In addition, rs4733781 and rs10956514 were included in our study because of their potential roles in conferring predisposition to TB. Finally, six SNPs in *SP110* and six SNPs in *ASAP1* were chosen for subsequent genotyping. Blood samples were collected from the cases and controls in EDTA-coated tubes and stored at –80°C. Genomic DNA was isolated from a 200 *μ*L aliquot of each blood sample using TIANamp Genomic DNA Kits (TIANGEN, Beijing, China). DNA degradation and contamination were monitored using 1% agarose gels, and DNA purity was checked using a NanoPhotometer® spectrophotometer (Implen, Westlake Village, CA, USA). The genotypes of polymorphic loci were detected using next-generation sequencing with the primers shown in Table S1. The SNP genotyping in the validation cohort was conducted by Sangon Biotech Co., Ltd. (Shanghai, China). High-throughput sequencing (Illumina Hi-seq 2000, San Diego, CA, USA) was performed for SNP genotyping of the candidate SNPs in *ASAP1* and *SP110*.

### 2.3. Statistical Analysis

EpiData 3.1 (EpiData Association, Odense, Denmark) and SPSS 20.0 (SPSS Inc., Chicago, IL, USA) software packages were used for statistical analysis. Categorical and continuous variables were compared using the *χ*^2^ test. Testing of the Hardy–Weinberg equilibrium (HWE) was used to determine whether the two groups were in genetic equilibrium. Logistic regression analysis was conducted to test the association between SNPs and TB. The distribution of allele frequencies, genotypes, the genetic dominant model, and the recessive model for each polymorphism were compared. Comparisons of frequencies between groups were presented as odds ratios (ORs) and 95% confidence intervals (CIs). We also used unconditional logistic regression analyses to calculate ORs and 95% CIs adjusted for sex and education level. Moreover, linkage disequilibrium (LD) analysis was conducted using Haploview 4.2 (Broad Institute, Cambridge, MA, USA). Statistical significance was set at the level of *p* < 0.05.

## 3. Results

### 3.1. Participant Characteristics

The demographic characteristics of all case and control participants are presented in Table 1. In total, 197 pulmonary TB patients (135 males and 62 females; mean age: 44.36 ± 15.62 years) and 217 healthy controls (114 males and 103 females; mean age: 44.95 ± 15.73 years) were included in this study. There were significant differences between the two groups in sex and education level (*p* = 0.003 and 0.005, respectively), but not for age, habitats (smoking and drinking), or marital status (*p* = 0.156, 0.093, 0.063, and 0.192, respectively).

### 3.2. HWE Test

In this study, six SNPs in *SP110* and six SNPs in *ASAP1* were selected for the HWE test.

The genotypic distributions of rs10956514, rs4733781, rs2033059, rs12680942, rs1017281, rs1469288, and rs17285138 in *ASAP1* were in accordance with the HWE among the study participants (Table S2).

The genotypic distributions of rs113579, rs9061, rs722555, rs3948464, rs11679983, rs1365576, and rs11556887 in *SP110* were also in accordance with the HWE among the pulmonary TB patients and healthy controls (Table S2).

### 3.3. Single SNP Associations

The genotypes and distributions of alleles are summarized in Tables 2 and 3. The impact of SNPs on susceptibility to TB was investigated using a case–control experimental design. All of the investigated SNPs were in agreement with the HWE in the study population. Among 12 SNPs that were successfully genotyped in *ASAP1* and *SP110*, only one SNP in the *SP110* gene (rs722555) was significantly associated with susceptibility to TB in the Mongolian population (Table 2).

The genotypes and allele frequencies of the six SNPs in *ASAP1* are summarized in Tables 2 and 3. Logistic regression analysis did not detect a significant association between *ASAP1* rs10956514 and the risk of TB (*p* = 0.889; allele OR = 1.07, 95% CI: 0.81–1.40). Similarly, no significant associations were observed for rs11774633, rs4733781, rs2033059, rs12680942, rs1469288, or rs17285138 in *ASAP1* (*p* > 0.05), whereas a trend of decreased risk of TB was observed for all of these variants.

For the *SP110* gene, we found an association between one SNP rs722555 and TB susceptibility. The T allele in rs722555 was significantly higher in TB patients compared with healthy individuals (OR 1.36, 95% CI 1.03–1.81, *p* = 0.03). Moreover, the CT genotype in rs722555 conferred a significantly increased risk, by 78%, compared with the wild-type CC genotype (OR 1.78, 95% CI 1.16–2.72, *p* = 0.008). Although the TT genotype in rs722555 appeared to indicate an increased risk of TB, this result was not statistically significant (OR 1.61, 95% CI 0.88–2.95, *p* = 0.121), which might be due to the limited sample size. There were no significant differences in distribution between the other alleles or genotypes and TB risk.

### 3.4. Associations between the Risk of TB and Genetic Models of SNPs

Additive, dominant, and recessive models of *ASAP1* and *SP110* gene polymorphisms were built to find the optimal genetic model.

As shown in Table 4, the rs722555 site in *SP110* was detected to confer an increased risk of TB in the dominant model (CT+TT vs. CC: OR, 1.74; 95% CI: 1.16–2.60; *p* = 0.007). We also found similar patterns in the recessive (CT+TT vs. CC: OR 1.16, 95% CI 0.67–2.02, *p* = 0.588) and additive (CT+TT vs. CC: OR 1.61, 95% CI 0.88–2.95, *p* = 0.121) models, albeit with no statistical significance. However, we did not observe any significant associations for the other selected SNPs in *ASAP1* and *SP110* in these models.

### 3.5. LD and Haplotype Analyses

**Figure 1:**
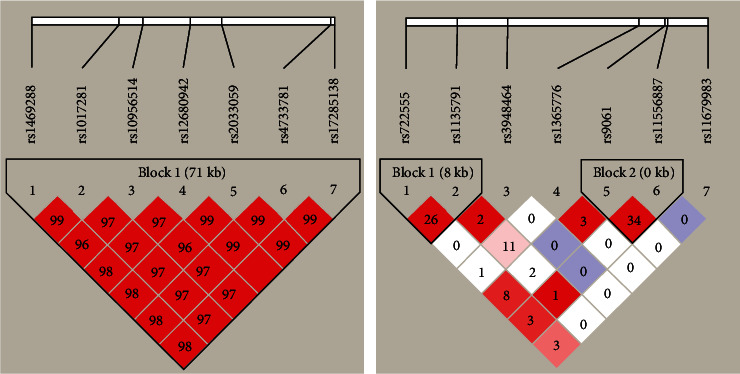
Linkage disequilibrium analysis of SNPs of *ASAP1* and *SP110* in the Mongolian population.

The LD was estimated by calculating the pairwise *r*^2^ coefficient.  Figure 1 shows the LD patterns for the cluster of six SNPs in *ASAP1* and six SNPs in *SP110* genotyped in the Mongolian population in China.

The LD patterns of SNPs in *ASAP1* are shown in Figure 1. Using a pairwise *r*^2^ > 0.8 as the threshold for strong LD, the six polymorphisms of *ASAP1* (rs1469288, rs10956514, rs12680942, rs2033059, rs4733781, and rs17285138) were in strong LD with one another, which suggests a strong recombination block. Haplotype analysis identified two haplotypes in this recombination block: AGGTCA and GAACAT (Table 5). When comparing the frequencies between cases and controls, there was no significant LD observed for these haplotypes (Table 5).

For *SP110*, we discovered two haplotype blocks (Figure 1), including four SNPs (block 1: rs722555 and rs1135791; block 2: rs9061 and rs11556887). As shown in Table 5, there were three haplotypes (CA, TA, and TG) in block 1, and three haplotypes (CG, TG, and TA) in block 2. However, when comparing the frequencies between cases and controls, there was no significant LD for these haplotypes.

## 4. Discussion

The association between *ASAP1* or *SP110* and susceptibility to TB has been investigated in various populations. The results from these studies are generally controversial [4, 6, 10–13, 22]. To further clarify the inconsistence, our study focused on a Mongolian population in China and revealed that one SNP in *SP110* (rs722555) rather than in *ASAP1* was associated with the risk of TB. Individuals with the CT and TT genotypes of rs722555 in *SP110* have an increased risk of pulmonary TB.

*ASAP1* gene encoding ASAP1 protein is a key regulator of membrane trafficking and the actin cytoskeleton [9, 23]; therefore, it plays important roles in many cellular functions including adhesion and motility, bone resorption, neurite outgrowth, and pathogen internalization by immune cells [15, 24]. The SNPs in *ASAP1* had been shown to be significantly associated with TB in a Russian population [5], or Han Chinese population [9], or Xinjiang Muslim population [14]. However, these SNPs seem not to be associated with pulmonary TB in a Mongolian population in our study, suggesting the potential effects of genetic diversity of human population.

In our study, the rs722555 SNP in the *SP110* gene was particularly notable, in which the CT genotype increased the risk of TB infection, with a 78% increase compared with the CC genotype (Table 2). Furthermore, individuals with T alleles were observed to be more susceptible to pulmonary TB than individuals with C alleles. These results are consistent with a previous study showing that rs722555 variation was associated with TB susceptibility in a Chongqing Han population [2], but in contrast to the results of studies which performed with a southern Chinese population [3] or the TB patients in Russia, which demonstrated that rs722555 was not significantly associated with TB (OR = 1.03, *p* = 0.46) [4]. The reasons for these discrepancies have yet to be investigated. There are likely individual differences in TB susceptibility between different populations. In the dominant model, similar trends were detected in regard to the increased risk of TB. We also found similar trends in the recessive model and the additive model, albeit with no statistical significance. Results of the LD analysis revealed that rs722555/rs1135791 and rs9061/rs11556887 were genetically linked.

*SP110*-encoded protein can inhibit the growth of intracellular pathogens by switching a cell death pathway from necrosis to apoptosis in infected macrophages [25]. It also regulates NF-*κ*B-mediated transcription [26], which is involved in immune responses, apoptosis, defense responses, and inflammatory responses [25]. Collectively, all these observations suggest that *SP110* may play potential roles in the TB susceptibility. However, how the SNPs in *SP110* affects the susceptibility to TB in the Mongolian population remains to be explored.

The results in this report demonstrate that the rs722555 SNP in the *SP110* gene is a risk factor for pulmonary TB susceptibility in the Mongolian population in China. SNPs in *ASAP1* had no association with TB susceptibility in this Mongolian population, although these SNPs may be associated with a reduced risk of TB in other populations. In conclusion, this study provides a new piece of evidence to support the importance of genetic variability of hosts in the pathogenesis of TB and may help to improve patient-specific clinical TB diagnosis or favor more suitable precautions against TB among high-risk individuals.

## 5. Conclusion

In conclusion, this study provides evidence to support the idea that genetic variability in the host could affect the susceptibility to TB. Our results indicate that the rs722555 SNP in *SP110* is a risk factor for TB susceptibility in the Mongolian population. In contrast, SNPs in *ASAP1* had no association with TB susceptibility in our Mongolian population, although these SNPs may be associated with a reduced risk of TB in this population. A large-scale GWAS therefore should be performed to obtain more solid evidence of whether these *ASAP1* SNPs are associated with TB in this ethnic minority. The results from our study may be beneficial for the assessment of genetic susceptibility factors and to improve the possible outcomes of TB infection in the Mongolian population.

## Figures and Tables

**Table 1:** Sociodemographic characterization among 197 tuberculosis patients and 217 healthy controls from Mongolian population.

Characteristics	Total^a^, *n* (%)	TB patients, *n* (%)	Healthy controls, *n* (%)	*p* value^c^
Age, mean ± SD (years)^b^	44.68 ± 15.67	44.36 ± 15.62	44.95 ± 15.73	0.156
*Sex*				
Male	240 (59.3)	126 (67.0)	114 (52.5)	0.003
Female	165 (40.7)	62 (33.0)	103 (47.5)	
*Smoking*				
Yes	116 (29.3)	60 (33.5)	56 (25.8)	0.097
No	280 (70.7)	119 (66.5)	161 (74.2)	
*Drinking*				
Yes	141 (35.6)	75 (41.9)	66 (30.4)	0.020
No	255 (64.4)	104 (58.1)	151 (69.6)	
*Education level*				
Primary school or lower	104 (26.2)	60 (33.3)	44 (20.3)	0.004
Junior school	195 (49.1)	74 (41.1)	121 (55.7)	
Senior high school and higher	98 (24.7)	46 (25.6)	52 (24.0)	
*Marital status*				
Married	313 (79.0)	145 (79.7)	168 (78.5)	0.805
Unmarried/divorced/widowed	83 (21.0)	37 (20.3)	46 (21.5)	

^a^Tuberculosis patients; ^b^healthy controls.

**Table 2:** Distribution frequency of SNPs of *ASAP1* and *SP110* gene in TB and healthy population.

Gene	SNP	Genotypes	TB (*n* = 197)	Control (*n* = 217)	*χ* ^2^	*p* value	Univariate logistic model	
							OR (95 CI)	*p* value
*ASAP1*	rs10956514	GG	59 (29.9)	61 (28.1)	0.234	0.889	**1.00 (ref)**	
		GA	107 (54.3)	119 (54.8)			0.93 (0.60, 1.45)	0.747
		AA	31 (15.7)	37 (17.1)			0.87 (0.48, 1.57)	0.637
	rs4733781	CC	61 (31.0)	61 (29.5)	0.432	0.806	**1.00 (ref)**	
		CA	104 (52.8)	118 (55.8)			0.88 (0.57, 1.37)	0.576
		AA	61 (31.0)	61 (29.5)			0.84 (0.47, 1.52)	0.568
	rs2033059	TT	61 (31.0)	61 (28.1)	0.432	0.806	**1.0 (ref)**	
		TC	104 (52.8)	118 (54.4)			0.88 (0.57, 1.37)	0.576
		CC	32 (16.2)	38 (17.5)			0.84 (0.47, 1.52)	0.568
	rs12680942	GG	62 (31.5)	61 (28.1)	0.636	0.727	**1.0 (ref)**	
		GA	104 (52.8)	118 (54.4)			0.87 (0.56, 1.35)	0.526
		AA	31 (15.7)	38 (17.5)	31 (15.7)	38 (17.5)	0.8 (0.44, 1.45)	0.466
	rs1469288	AA	60 (30.5)	60 (27.6)	0.496	0.780	**1.0 (ref)**	
		AG	106 (53.8)	119 (54.9)			0.89 (0.57, 1.39)	0.609
		GG	31 (15.7)	38 (17.5)	31 (15.7)	38 (17.5)	0.82 (0.45, 1.48)	0.502
	rs17285138	AA	62 (31.5)	61 (28.1)	0.636	0.727	**1.0 (ref)**	
		AT	104 (52.8)	118 (54.4)			0.87 (0.56, 1.35)	0.526
		TT	31 (15.7)	38 (17.5)			0.8 (0.44, 1.45)	0.466

*SP110*	rs1135791	AA	137 (69.5)	156 (71.9)	1.696	0.428	**1.0 (ref)**	
		AG	55 (27.9)	59 (27.2)			1.06 (0.69, 1.64)	0.787
		GG	5 (2.5)	2 (0.9)			2.85 (0.54, 14.91)	0.216
	rs9061	CC	135 (68.5)	139 (64.1)	1.002	0.606	**1.0 (ref)**	
		CT	58 (29.4)	72 (33.2)			0.83 (0.55, 1.26)	0.382
		TT	4 (2.0)	6 (2.8)			0.69 (0.19, 2.49)	0.567
	rs722555	CC	61 (31)	95 (43.8)	7.327	0.026	**1.0 (ref)**	
		CT	106 (53.8)	93 (42.9)			**1.78 (1.16, 2.72)**	**0.008**
		TT	30 (15.2)	29 (13.4)			1.61 (0.88, 2.95)	0.121
	rs3948464	GG	197 (100)	213 (98.2)	3.667	0.074	**NA**	
		GA	0	4 (1.8)			**1.0 (ref)**	
		AA	0	0	0	0	**NA**	
	rs11679983	GG	181 (91.9)	195 (89.9)	0.504	0.296	**1.0 (ref)**	
		GA	16 (8.1)	22 (10.1)			0.78 (0.4, 1.54)	0.479
		AA	0	0			**NA**	
	rs1365776	TT	150 (76.1)	167 (77)	0.985	0.619	**1.0 (ref)**	
		TC	46 (23.4)	47 (21.7)			2.94 (0.29, 29.27)	0.359
		CC	1 (0.5)	3 (1.4)			2.69 (0.28, 26.18)	0.393
	rs11556887	GG	166 (84.3)	188 (86.6)	0.469	0.293	**1.0 (ref)**	
		GA	31 (15.7)	29 (13.4)			1.21 (0.7, 2.09)	0.494
		AA	0	0			**NA**	

Data are presented as *n* (%). SNP: single-nucleotide polymorphism; TB: tuberculosis; OR: odds ratio; CI: confidence intervals; ref: reference; NA: not applicable. *p* values below 0.05 are highlighted in bold.

**Table 3:** The distribution of alleles of SNPs in the *ASAP1* and *SP110* genes in TB and healthy control population.

Gene	SNP	Alleles	TB, *N* (%)	Control, *N* (%)	Univariate logistic model	
					OR (95 CI)	*p* value
*ASAP1*	rs10956514	G	225 (57.1)	241 (55.5)	1.0 (ref)	
		A	169 (42.9)	193 (44.5)	0.94 (0.71, 1.23)	0.648
	rs4733781	C	226 (57.4)	240 (55.3)	1.0 (ref)	
		A	168 (42.6)	194 (44.7)	0.92 (0.7, 1.21)	0.551
	rs2033059	T	226 (57.4)	240 (55.3)	1.0 (ref)	
		C	168 (42.6)	194 (44.7)	0.92 (0.7, 1.21)	0.551
	rs12680942	G	228 (57.9)	240 (55.3)	1.0 (ref)	
		A	166 (42.1)	194 (44.7)	0.9 (0.68, 1.19)	0.457
	rs1469288	A	226 (57.4)	239 (55.1)	1.0 (ref)	
		G	168 (42.6)	195 (44.9)	0.91 (0.69, 1.2)	0.507
	rs17285138	A	228 (57.9)	240 (55.3)	1.0 (ref)	
		T	166 (42.1)	194 (44.7)	0.9 (0.68, 1.19)	0.457

*SP110*	rs1135791	A	329 (83.5)	371 (85.5)	1.0 (ref)	
		G	65 (16.5)	63 (14.5)	1.16 (0.8, 1.7)	0.431
	rs9061	C	328 (83.2)	350 (80.6)	1.0 (ref)	
		T	66 (16.8)	84 (19.4)	0.84 (0.59, 1.2)	0.332
	rs722555	C	228 (57.9)	283 (65.2)	1.0 (ref)	
		T	166 (42.1)	151 (34.8)	**1.36 (1.03, 1.81)**	**0.030**
	rs3948464	G	394 (100)	430 (99.1)	1.0 (ref)	
		A	0 (0)	4 (0.9)	NA	NA
	rs11679983	G	378 (95.9)	412 (94.9)	1.0 (ref)	
		A	16 (4.1)	22 (5.1)	0.79 (0.41, 1.53)	0.490
	rs1365776	T	346 (87.8)	381 (87.8)	1.0 (ref)	
		C	48 (12.2)	53 (12.2)	1 (0.66, 1.51)	0.990
	rs11556887	G	363 (92.1)	405 (93.3)	1.0 (ref)	
		A	31 (7.9)	29 (6.7)	1.19 (0.7, 2.02)	0.511

Data are presented as *n* (%). SNP: single-nucleotide polymorphism; TB: tuberculosis; OR: odds ratio; CI: confidence intervals; NA: not applicable. *p* values below 0.05 are highlighted in bold.

**Table 4:** Analysis of the inheritance models of *ASAP1* and *SP110* polymorphism associated with tuberculosis.

Gene	SNP	Model	Genotype	TB, *N* (%)	Control, *N* (%)	Univariate logistic model	
						OR (95 CI)	*p* value
*ASAP1*	rs10956514	Dominant	G/G G/A-A/A	59 (29.9) 138 (70.1)	61 (28.1) 156 (71.9)	0.91 (0.60, 1.40)	0.681
		Recessive	G/G-G/A A/A	166 (84.3) 31 (15.7)	180 (82.9) 37 (17.1)	0.91 (0.54, 1.53)	0.718
		Additive	G/G A/A	59 (29.9) 31 (15.7)	61 (28.1) 37 (17.1)	0.87 (0.48, 1.57)	0.637
	rs4733781	Dominant	C/C A/C-A/A	61 (31.0) 136 (69.0)	61 (28.1) 156 (71.9)	0.87 (0.57, 1.33)	0.525
		Recessive	C/C-A/C A/A	165 (83.8) 32 (16.2)	179 (82.5) 38 (17.5)	0.91 (0.55, 1.53)	0.731
		Additive	C/C A/A	61 (31.0) 32 (16.2)	61 (28.1) 38 (17.5)	0.84 (0.47, 1.52)	0.568
	rs2033059	Dominant	T/T C/T-C/C	61 (31.0) 136 (69.0)	61 (28.1) 156 (71.9)	0.87 (0.57, 1.33)	0.525
		Recessive	T/T-C/T C/C	165 (83.8) 32 (16.2)	179 (82.5) 38 (17.5)	0.91 (0.55, 1.53)	0.731
		Additive	T/T C/C	61 (31.0) 32 (16.2)	61 (28.1) 38 (17.5)	0.84 (0.47, 1.52)	0.568
	rs12680942	Dominant	G/G A/G-A/A	62 (31.5) 135 (68.5)	61 (28.1) 156 (71.9)	0.85 (0.56, 1.30)	0.455
		Recessive	G/G-A/G A/A	166 (84.3) 31 (15.7)	179 (82.5) 38 (17.5)	0.88 (0.52, 1.48)	0.628
		Additive	G/G A/A	62 (31.5) 31 (15.7)	61 (28.1) 38 (17.5)	0.80 (0.44, 1.45)	0.466
	rs1469288	Dominant	A/A G/A-G/G	60 (30.5) 137 (69.5)	60 (27.6) 157 (72.4)	0.87 (0.57, 1.33)	0.530
		Recessive	A/A-G/A G/G	166 (84.3) 31 (15.7)	179 (82.5) 38 (17.5)	0.88 (0.52, 1.48)	0.628
		Additive	A/A G/G	60 (30.5) 31 (15.7)	60 (27.6) 38 (17.5)	0.82 (0.45, 1.48)	0.502
	rs17285138	Dominant	A/A T/A-T/T	62 (31.5) 135 (68.5)	61 (28.1) 156 (71.9)	0.85 (0.56, 1.30)	0.455
		Recessive	A/A-T/A T/T	166 (84.3) 31 (15.7)	179 (82.5) 38 (17.5)	0.88 (0.52, 1.48)	0.628
		Additive	A/A T/T	62 (31.5) 31 (15.7)	61 (28.1) 38 (17.5)	0.80 (0.44, 1.45)	0.466

*SP110*	rs1135791	Dominant	A/A A/G-G/G	137 (69.5) 60 (30.5)	156 (71.9) 61 (28.1)	1.12 (0.73, 1.71)	0.600
		Recessive	A/A-A/G G/G	192 (97.5) 5 (2.5)	215 (99.1) 2 (0.9)	2.80 (0.54, 14.60)	0.222
		Additive	A/A G/G	137 (69.5) 5 (2.5)	156 (71.9) 2 (0.9)	2.85 (0.54, 14.91)	0.216
	rs9061	Dominant	C/C C/T-T/T	135 (68.5) 62 (31.5)	139 (64.1) 78 (35.9)	0.82 (0.54, 1.23)	0.337
		Recessive	C/C-C/T T/T	193 (98.0) 4 (2.0)	211 (97.2) 6 (2.8)	0.73 (0.20, 2.62)	0.628
		Additive	C/C T/T	135 (68.5) 4 (2.0)	139 (64.1) 6 (2.8)	0.69 (0.19, 2.49)	0.567
	rs722555	Dominant	C/C C/T-T/T	61 (31.0) 136 (69.0)	95 (43.8) 122 (56.2)	**1.74 (1.16, 2.60)**	**0.007**
		Recessive	C/C-C/T T/T	167 (84.8) 30 (15.2)	188 (86.6) 29 (13.4)	1.16 (0.67, 2.02)	0.588
		Additive	C/C T/T	61 (31.0) 30 (15.2)	95 (43.8) 29 (13.4)	1.61 (0.88, 2.95)	0.121
	rs3948464	Dominant	G/G G/A-AA	197 (100.0) 0 (0.0)	213 (98.2) 4 (1.8)	**NA**	
		Recessive	G/G-G/A AA	197 (100.0) 0 (0.0)	217 (100.0) 0 (0.0)	**NA**	
		Additive	G/G AA	197 (100.0) 0 (0.0)	213 (98.2) 0 (0.0)	**NA**	
	rs11679983	Dominant	G/G G/A-AA	181 (91.9) 16 (8.1)	195 (89.9) 22 (10.1)	0.78 (0.40, 1.54)	0.479
		Recessive	G/G-G/A AA	197 (100.0) 0 (0.0)	217 (100.0) 0 (0.0)	**NA**	
		Additive	G/G AA	181 (91.9) 0 (0.0)	195 (89.9) 0 (0.0)	**NA**	
	rs1365776	Dominant	T/T C/T-C/C	150 (76.1) 47 (23.9)	167 (77.0) 50 (23.0)	1.05 (0.66, 1.65)	0.845
		Recessive	T/T-C/T C/C	196 (99.5) 1 (0.5)	214 (98.6) 3 (1.4)	0.36 (0.04, 3.53)	0.383
		Additive	T/T C/C	150 (76.1) 1 (0.5)	167 (77.0) 3 (1.4)	0.37 (0.04, 3.61)	0.393
	rs11556887	Dominant	GG GA-AA	166 (84.3) 31 (15.7)	188 (86.6) 29 (13.4)	1.21 (0.70, 2.09)	0.494
		Recessive	G/G-GA A/A	197 (100.0) 0 (0.0)	217 (100.0) 0 (0.0)	**NA**	
		Additive	GG A/A	166 (84.3) 0 (0.0)	188 (86.6) 0 (0.0)	**NA**	

Data are presented as *n* (%). SNP: single-nucleotide polymorphism; TB: tuberculosis; OR: odds ratio; CI: confidence intervals; NA: not applicable. *p* values below 0.05 are highlighted in bold.

**Table 5:** The haplotypes analysis of *ASAP1* and *SP110* gene and TB in Mongolian population.

Gene	SNPs	Group	Haplotype	TB case_F	Healthy control_F	OR	95% CI	*p* value
*ASAP1*	rs1469288 rs10956514 rs12680942 rs2033059 rs4733781 rs17285138	Active tuberculosis vs. heath controls	AGGTCA	111 (0.563)	119 (0.551)	0.94	0.64-1.39	0.758
			GAACAT	83 (0.419)	97 (0.445)	1.11	0.75-1.64	0.599

*SP110*	rs7222555 rs1135791	Active tuberculosis vs. heath controls	CA	113 (0.572)	141 (0.649)	1.38	0.93-2.05	0.112
			TA	52 (0.263)	45 (0.206)	0.73	0.46-1.15	0.175
			TG	31 (0.159)	31 (0.142)	0.89	0.52-1.53	0.680
	rs9061 rs11556887	Active tuberculosis vs. heath controls	CG	164 (0.832)	175 (0.806)	0.84	0.51-1.39	0.492
			TG	17 (0.089)	27 (0.127)	1.50	0.79-2.85	0.211
			TA	16 (0.079)	15 (0.067)	0.84	0.40-1.75	0.641

Note: SNP: single-nucleotide polymorphism; TB: tuberculosis; OR: odds ratio; CI: confidence intervals; vs., versus.

## Data Availability

All relevant data are available within the manuscript and its supporting information files.

## Abbreviations

Mtb: Mycobacterium tuberculosis
TB: Tuberculosis
SNP: Single-nucleotide polymorphisms
ASAP1: Ankyrin repeat and PH domain 1
SP110: Speckled 110
OR: Odds ratios
CI: Confidence intervals
LPS: Lipopolysaccharide
PTB: Pulmonary tuberculosis
HWE: Hardy-Weinberg equilibrium
LD: Linkage disequilibrium
LTBI: Latent tuberculosis infection.

## Supplementary Materials

Supplementary 1 Supplementary Table 1: primers for SNPs in *ASAP1* and *SP110.*

Supplementary 2 Supplementary Table 2: analysis of SNP of TB acceptability associated genes using H-WE.
