# Supplementary material for: Polymorphisms in the ASAP1 and SP110 Genes and Its Association with the Susceptibility to Pulmonary Tuberculosis in a Mongolian Population
Source: J Immunol Res. 2022 Sep 20;2022:2713869. doi: 10.1155/2022/2713869 (PMC9557252; doi:10.1155/2022/2713869)
Supplement: Supplementary 1 — Supplementary Table 1: primers for SNPs in ASAP1 and SP110. [file 2713869.f1.docx]

Supplementary Table 1. primers for SNPs in *ASAP1* and *SP110*

| 基因 | SNP | 5'-Forward primer-3' | 5'-Reverse primer-3' |
| --- | --- | --- | --- |
| *ASAP1* | rs10956514 | AATGCACTTTTAAGAGCCTCATGTTTTA | ATACTTAGAAATGCCCGCACACG |
| *ASAP1* | rs4733781 | GAAAACCCATGAAAATTTTTAAGCAGGG | GTCATGCTTTGTCAGTGATTACAGAGA |
| *ASAP1* | rs2033059 | GATTTCTTCCCTGCAAAACTGGAAA | CAGCTCAGGTCATGAATTTTGTGAAC |
| *ASAP1* | rs12680942 | CTTATGTTGCTGCTATAAAGACCCAGA | GTCAAATGTAAGGGTGGTTTTGTGAC |
| *ASAP1* | rs1017281 | AGAAGGCACTCAAGAATTCATCTAGCT | CCCCTTTGAAAAAGAGAGAAACTCAAG |
| *ASAP1* | rs1469288 | TCTACTCTATCAGCTGGGCCAAAG | CACACTGCTGAAAAATCTGGTAAGAC |
| *ASAP1* | rs17285138 | TCGGCCAAAACAAAATGATCTTATCAC | CAGGTTGACACTAATCATGGATGC |
| *SP110* | rs1135791 | TTCTCCTTCAAGGATTTAAACCTGTCA | GCCACAATTAGGATGAAGAGGTTCAG |
| *SP110* | rs9061 | TTTGTATTGCTCAGTTCTTTTTCCCAG | CACTTTTAACATTCACTCTTCTCCCTGA |
| *SP110* | rs722555 | AGCTTAAAAGAGACATAGGGACAGGAGA | CCCAAACCCAGACACTTCATAGGAT |
| *SP110* | rs3948464 | ACAACCACTGTCACATCAACAGATC | GTGGTGGATAAGGTGACTCAAAGG |
| *SP110* | rs11679983 | CTTCCCAAGTACCTCTAGTCATTTAC | CTGCAGGGTGGAGTTCAACTATT |
| *SP110* | rs1365776 | AGAATAAAATTCAACCCTCCACAGCTTG | TTCTAGCAATGTGGTTTGAAGACCAAA |
| *SP110* | rs11556887 | ACTCAGGATCTCATCGCTTTGCT | ATGTATTAAGAAATCCCCTTCCCCAAG |
