# Supplementary material for: Polymorphisms in the ASAP1 and SP110 Genes and Its Association with the Susceptibility to Pulmonary Tuberculosis in a Mongolian Population
Source: J Immunol Res. 2022 Sep 20;2022:2713869. doi: 10.1155/2022/2713869 (PMC9557252; doi:10.1155/2022/2713869)
Supplement: Supplementary 2 — Supplementary Table 2: analysis of SNP of TB acceptability associated genes using H-WE. [file 2713869.f2.docx]

Supplementary Table 2. Analysis of SNP of TB acceptability Associated Genes using H-WE

| Genes | SNP | CHR | Position | Region | H-WE | H-WE-Case | H-WE-control |
| --- | --- | --- | --- | --- | --- | --- | --- |
| *ASAP1* | rs10956514 | 8 | 203337 | intron | 0.059 | 0.12 | 0.10 |
| *ASAP1* | rs4733781 | 8 | 159328 | intron | 0.060 | 0.29 | 0.14 |
| *ASAP1* | rs2033059 | 8 | 184879 | intron | 0.060 | 0.26 | 0.14 |
| *ASAP1* | rs12680942 | 8 | 192062 | intron | 0.060 | 0.24 | 0.14 |
| *ASAP1* | rs1017281 | 8 | 208882 | intron | 0.019 | 0.11 | **0.04** |
| *ASAP1* | rs1469288 | 8 | 229443 | intron | 0.051 | 0.16 | 0.11 |
| *ASAP1* | rs17285138 | 8 | 158399 | intron | 0.060 | 0.24 | 0.14 |
| *SP110* | rs1135791 | 2 | 48170 | coding | 0.270 | 0.85 | 0.15 |
| *SP110* | rs9061 | 2 | 13335 | intron | 0.230 | 0.43 | 0.35 |
| *SP110* | rs722555 | 2 | 56930 | 3’UTR | 0.720 | 0.14 | 0.41 |
| *SP110* | rs3948464 | 2 | 39731 | coding | 0.920 | 1.00 | 0.89 |
| *SP110* | rs11679983 | 2 | 6144 | 5’UTR | 0.320 | 0.55 | 0.43 |
| *SP110* | rs1365776 | 2 | 17736 | intron | 0.320 | 0.20 | 0.88 |
| *SP110* | rs11556887 | 2 | 12769 | intergentic | 0.110 | 0.23 | 0.29 |

SNP, single nucleotide polymorphisms; H-WE, hardy Weinberg Equilibrium.
